# Supplementary material for: Inhibition of aquaporin-3 in macrophages by a monoclonal antibody as potential therapy for liver injury
Source: Nat Commun. 2020 Nov 9;11:5666. doi: 10.1038/s41467-020-19491-5 (PMC7653938; doi:10.1038/s41467-020-19491-5)
Supplement: Supplementary file 1 — Supplementary Information [file 41467_2020_19491_MOESM1_ESM.pdf]

Supplementary Method

Mouse model of AOM-induced liver injury

AOM (azoxymethane, Sigma, 10 μg/g body weight) or vehicle (saline) was injected intraperitoneally once weekly for 6 weeks and mice were sacrificed at 15 weeks. For acute injury, mice were received an intraperitoneal injection of AOM (30 μg/g body weight) or vehicle (saline). Mice were sacrificed at 24 hours after the final AOM injection. In some studies, anti-AQP3 mAb (mAb-J, 10 mg/kg, PBS) or mouse monoclonal antibody (as control IgG) was administered intravenously 1 day before each AOM injection.

Mouse model of TAA-induced liver injury

WT or AQP3$^{-/-}$ mice received an intraperitoneal injection of TAA (thioacetamide, Nacalai, 200 μg/g body weight) or vehicle (saline). Mice were sacrificed at 24 or 72 hours after TAA injection. In some studies, anti-AQP3 mAb (mAb-J, 10 mg/kg body weight, PBS) or mouse monoclonal antibody (as control IgG) was administered intravenously (10 mg/kg weight) 1 day before TAA injection.

Water permeability

Osmotic water permeability was measured in human AQP3-expressing CHO-K1 cells. Cells were incubated with mAb (1 μg/ml) for 1 hr and stained with calcein AM (10 μM, 15 min, Invitrogen). Osmotic cell shrinkage was induced by addition of an equal volume of 600 mM sucrose in buffer (PBS), and calcein fluorescence quenching was recorded using a plate reader (Ex, 492nm, Em, 525 nm, SpectraMax i3x; Molecular Devices). Vmax was calculated using GraphPad Prism8.

Immunofluorescence of human liver tissues

Frozen human liver sections were obtained from Novus Biologicals (#NBP2-59069). Sections were fixed with cold acetone, and immunostained with anti-AQP3 (Millipore, #AB3276, 1:200) and anti-CD68 (Proteintech, #66231-2-lg, 1:100), following by anti-rabbit secondary antibody (Sigma, #C2306, 1:200) and anti-mouse secondary antibody (Invitrogen, A11029).

Supplementary table 1. List of anti-AQP3 mAb with CDR sequence of the heavy chains

sample#	*abbreviation*	*CDR1*	*CDR2*	*CDR3*
A_1d002	*A*	*GYTFTDYEMH*	*GVDPETGGTGYNQKFRG*	*ARHGGSFYAMDY*
B_1d004	*B*	*GFTFSSYGMS*	*TISRGSIYTYYPDSVKG*	*ARLSLYDYDGARYTMDY*
C_1d007	*C*	*GYNFKSYGIS*	*EIYPGSGNTYYNEKLKG*	*ARTYGYDSFPWFAY*
D_2d005	*D*	*GYTFTDYEMH*	*GIDPETGGTGYNQKFKG*	*TRHGSYAMDY*
E_1d034	*E*	*GYTFTDYEMH*	*GIDPESGGTGYNQKFKG*	*TRSGYYGSPLLDY*
F_1d039	*F*	*GYTFTDYYIN*	*WIFPGSGSTYYNEKFKG*	*ADYGSSYRYFDV*
G_2d039	*G*	*GYTFTDYEMH*	*GIDPETGGTAYNQKFKG*	*TRWGAITSFVALRGFAY*
H_1d020	*H*	*GFTFSSYGMS*	*TISRRSIYTYYPDSVQG*	*ARLSLYDYDGARYTMDY*
J_2d002	*J*	*GYTFTSYWMH*	*NINPSNGGTNYNEKFKS*	*ARGGIYYGNYDYYAMDY*
K_2d035	*K*	*GYAFTNYLIE*	*VINPGSGGTNYNEKFKG*	*ARWGFYYAMDY*

Supplementary table 2. Binding pattern of mAbs to peptide mutants

		mAb-C	mAb-E	mAb-H	mAb-J
4	C-SGPNGTAGUFATYPS-NH2	0.372	0.193	0.385	0.308
5	C-ATYPSGHLDM-NH2	**0.582**	**0.227**	**0.491**	**0.418**
5a	C-TYPSGHLDM-NH2	0.591	0.168	0.502	0.409
5b	C-YPSGHLDM-NH2	0.630	0.164	0.310	0.417
5c	C-PSGHLDM-NH2	0.488	0.201	0.288	0.334
5d	C-SGHLDM-NH2	0.379	0.182	0.350	0.335
5e	C-GHLDM-NH2	0.291	0.203	0.305	0.280
5f	C-ATYPSGHLD-NH2	0.597	0.135	0.333	0.447
5g	C-ATYPSGHL-NH2	0.529	0.178	0.383	0.319
5h	C-ATYPSGH-NH2	0.384	0.087	0.349	0.297
5i	C-ATYPSG-NH2	0.326	0.115	0.338	0.305
5j	C-TAGIFATYPSGHLDM-NH2	0.577	0.135	0.484	0.450
5k	C-AGIFATYPSGHLDM-NH2	0.627	0.134	0.471	0.437
5l	C-GIFATYPSGHLDM-NH2	0.598	0.117	0.532	0.435
5m	C-IFATYPSGHLDM-NH2	0.635	0.127	0.498	0.445
5n	C-FATYPSGHLDM-NH2	0.602	0.136	0.495	0.468

Peptide 5 (148 to 157 in loop C) and fifteen kinds of peptide mutants were synthesized as described above. The microtiter wells were coated with each peptide (1mg/ml, 50 μl, 4℃, overnight). After washing, the well was incubated with each antibody (1 μg/ml, 1hr) and anti-mouse IgG HRP conjugate (Sigma A8924, 1:10,000). The wells were developed with TMB solution and then stopped with 2N sulfuric acid. The absorbance at 450 nm was measured.

Supplementary table 3. Key residues for four mAbs

antibody	*residues*
mAb-C	*YPSGHLD*
mAb-E	*ATYPSGHLDM*
mAb-H	*TYPSGHLDM*
mAb-J	*YPSGHLD*

Supplementary table 4. Primers for qRT-PCR analysis (mouse)

Gene	*Forward/ Reverse*	*Sequence*
18s	*F*	*GAGGCCCTGTAATTGGAATGAG*
	R	*GCAGCAACTACTTTAATATACGCTATTGG*
HPRT1	*F*	*GAGGAGTCCTGTTGATGTTGCCAG*
	R	*GGCTGGCCTATAGGCTCATAGTGC*
TNF-alpha	*F*	*CCCTCACACTCAGATCATCTTCT*
	R	*GCTACGACGTGGGCTACAG*
CCl2	*F*	*GTGATGGAGGGGGTCAGGA*
	R	*GGGATGGGACAGCCTAAACT*
alpha-SMA	*F*	*GTTCAGTGGTGCCTCTGTCA*
	R	*ACTGGGACGACATGGAAAAG*
iNOS	*F*	*GTTCTCAGCCCAACAATACAAGA*
	R	*GTGGACGGGTCGATGTCAC*
Collagen1 alpha	*F*	*GATGACGTGCAATGCAATGAA*
	R	*CCCTCGACTCCTACATCTTCTGA*
TIMP-1	*F*	*TCTCTAGGAGCCCCGATCTG*
	R	*GCATGGACATTTATTCTCCACTGT*
MMP9	*F*	*GGACCCGAAGCGGACATTG*
	R	*CGTCGTCGAAATGGGCATCT*
TGF-beta1	*F*	*CTCCCGTGGCTTCTAGTGC*
	R	*GCCTTAGTTTGGACAGGATCTG*
CD44	*F*	*TGCAGGTATGGGTTCATAGAAGG*
	R	*GTGTTGGACGTGACGAGGA*
CD24	*F*	*TTCTGGCACTGCTCCTACC*
	R	*GCGTTACTTGGATTTGGGGAA*
IL-6	*F*	*TAGTCCTTCCTACCCCAATTTCC*
	R	*TTGGTCCTTAGCCACTCCTTC*
AQP3	*F*	*GCTTTTGGCTTCGCTGTCAC*
	R	*TAGATGGGCAGCTTGATCCAG*
ARG1	*F*	*CCACAGTCTGGCAGTTGGAAG*
	R	*GGTTGTCAGGGGAGTGTTGATG*
Emr1	*F*	*GGAGGACTTCTCCAAGCCTATT*
	R	*AGGCCTCTCAGACTTCTGCTT*
Desmin	*F*	*GCGTGACAACCTGATAGACG*
	R	*TGGATTTCCTCCTGTAGTTTGG*
Albumin	*F*	*TGACCCAGTGTTGTGCAGAG*
	R	*TTCTCCTTCACACCATCAAGC*

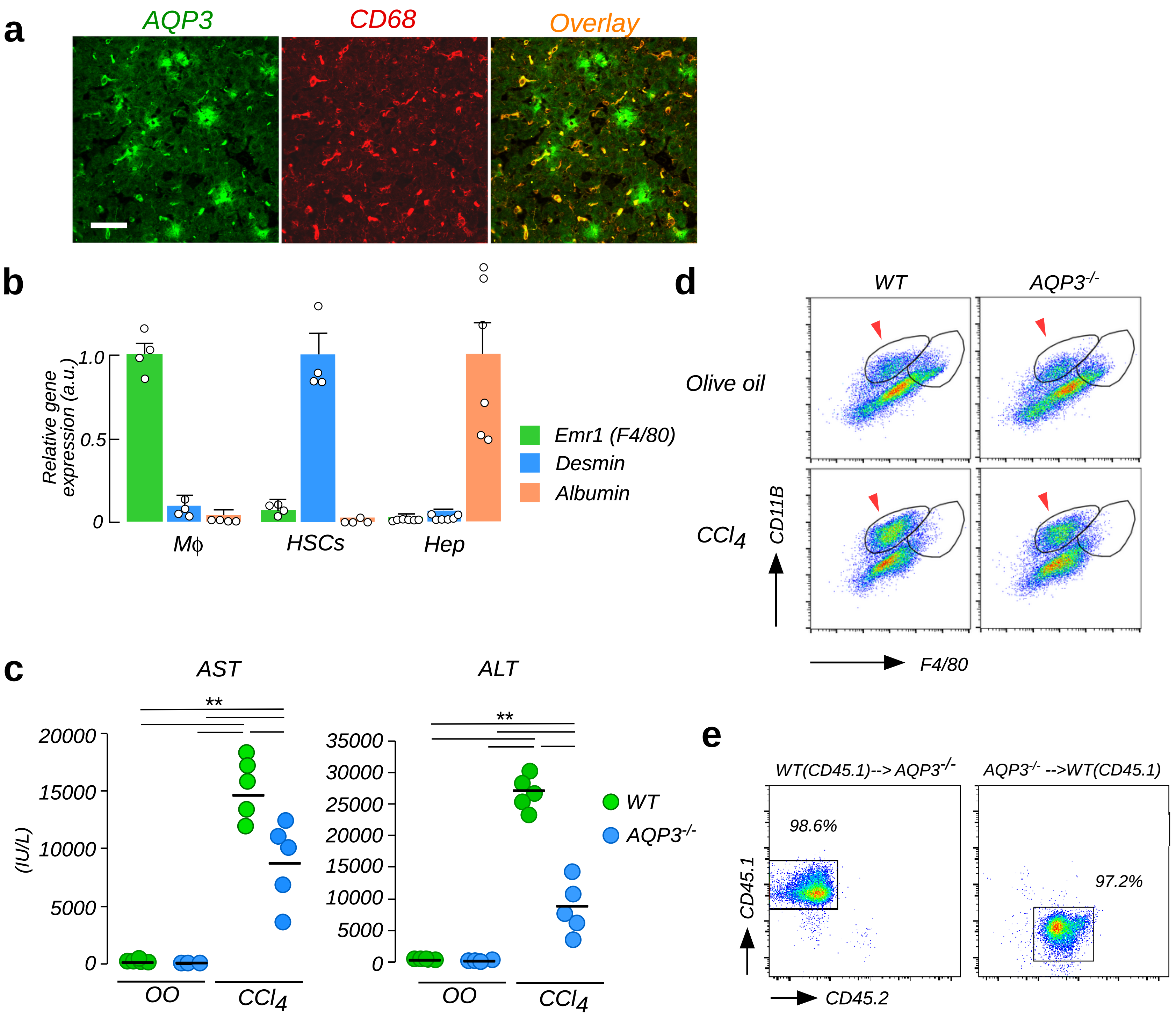

Supplementary Figure 1

a. Immunofluorescence with anti-AQP3 (FITC, green) and anti-CD68 (cy3, red) on frozen normal human liver. Bar. 100 µm. **b.** Macrophages (Mϕ), HSCs, and hepatocytes were isolated from WT liver. mRNA expression of Emr1 (F4/80, for macrophages), desmin (for HSCs), and albumin (for hepatocytes) by real-time RT-PCR (mean ± SE, n=6 for hepatocytes, n=4 for macrophage and HSC biologically independent samples), demonstrating high purity of each cell population. **c.** Serum AST and ALT corresponding to Fig. 1c-1f. (SE, n=5 mice/group, $^{**}p < 0.01$ by two-way ANOVA with Tukey's multiple comparisons test). **d.** Representative dot plots by flow cytometry analysis of hepatic cells stained with anti-CD11B and anti-F4/80, corresponding to Fig. 1d. Arrowheads indicate CD11B^{high} F4/80^{inter}. **e.** Mice (WT or AQP3$^{-/-}$, age 8-10 weeks) were gamma-irradiated (900 rad) and then injected intravenously with bone marrow cells from WT or AQP3$^{-/-}$ mice, corresponding to Fig. 1g. Chimerism was confirmed using C57BL/6 CD45.1 congenic mice in which spleen cells at 60 days after of bone marrow transfer were stained with CD45.1-PE-Cy7 and CD45.2-APC, and analyzed by FACS. More than 95% of the recipient cells were replaced by donor cells. Source data, including exact p values, are provided as a Source Data file.

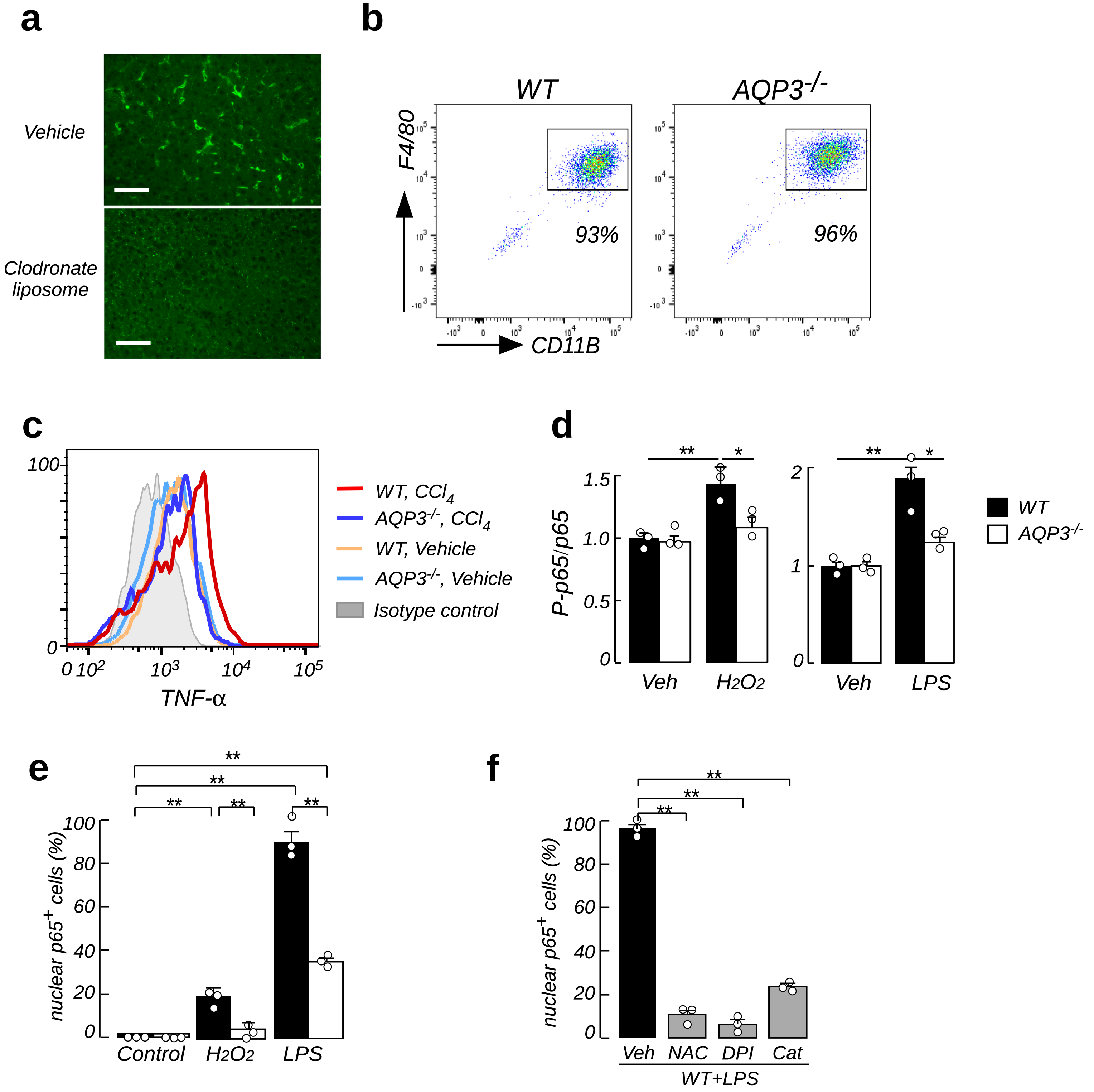

Supplementary Figure 2

a. Immunofluorescence with anti-F4/80 antibody (FITC, green) in WT liver without or with clodronate liposome injection, corresponding to Fig. 2a-2c. Bar, 100 μm. **b.** Representative dot plots by flow cytometry analysis on bone marrow-derived macrophages from WT and AQP3$^{-/-}$ mice stained with anti-CD11B and anti-F4/80 antibodies, demonstrating high purity of macrophages. **c.** FACS analysis of TNF-α expression in CD11B^{+} F4/80^{+} hepatic macrophages after CCl_4 injection. MFI of TNF-α corresponding to Fig. 2f. **d.** The ratio of P-p65 to p65, from data as in Fig. 2h (mean±SE, n=3 biologically independent samples, *$p < 0.05$, p** < 0.01 by two-way ANOVA with Tukey's multiple comparisons test). **e.** Naive macrophages were incubated with H_2O_2 (300 μM) or LPS (100 ng/ml) for 30 min. Numbers of p65-positive macrophages with nuclear staining (mean±SE, n=3 biologically independent samples, > 100 cells from over two different fields from one sample, **$p < 0.01$ by two-way ANOVA with Tukey's multiple comparisons test). **f.** Naive macrophages were incubated with NAC (50 μM), DPI (20 μM), or catalase (2000 U/ml), and stimulated with LPS (100 ng/ml, 30 min). Numbers of p65-positive macrophages with nuclear staining (mean±SE, n=3 biologically independent samples, > 100 cells from over two different fields from one sample, **$p < 0.01$ by one-way ANOVA). Source data, including exact p values, are provided as a Source Data file.

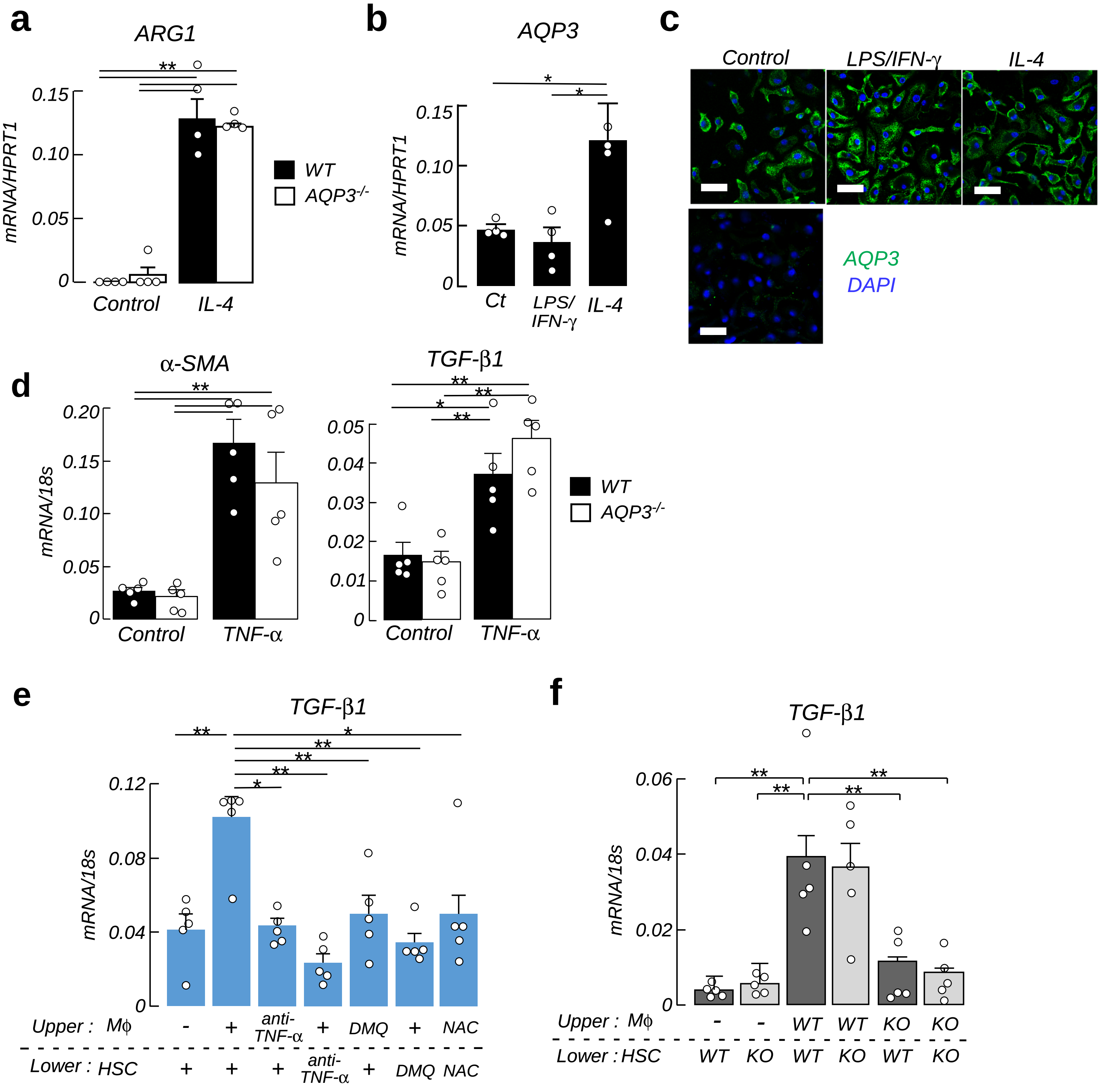

Supplementary Figure 3

a. Naive macrophages from WT or AQP3$^{-/-}$ mice were treated with IL-4 (20 ng/ml) for 24 hrs. Expression of ARG-1 mRNA shown as the ratio to HPRT1 (mean±SE, n=4 biologically independent samples, **p < 0.01). **b. c.** Naive WT macrophages were treated with LPS/IFN-γ (1 ng/ml for LPS, 10 ng/ml for IFN-γ) or IL-4 (20 ng/ml) for 24 hrs. **b.** Expression of AQP3 mRNA shown as the ratio to HPRT1 (mean±SE, n=4 biologically independent samples, *p < 0.05). **c.** Representative immunofluorescence of AQP3 (green, AQP3; blue, DAPI). Bar, 20 µm. **d.** Naive HSC from WT or AQP3$^{-/-}$ mice were treated with TNF-α (100 ng/ml) for 24 hrs. Expression of α-SMA and TGF-β1 mRNA shown as the ratio to 18s (mean±SE, n=5 biologically independent samples, *p < 0.05, **p < 0.01). Statistical analysis for Sup Fig. 3a-d was performed by two-way ANOVA. **e.** WT HSCs or macrophages were incubated with anti-TNF-α (1 µg/ml), DHMEQ (1 µg/ml), or NAC (50 µM), and then co-cultured, corresponding to Fig. 3e. Macrophages were isolated from CCl$_4$-injected WT mice. mRNA expression of TGF-β1 in HSCs (mean±SE, n=5 biologically independent samples, *p <0.05, **p <0.01 by one-way ANOVA Dunnett's multiple comparisons test). **f.** HSCs from WT or AQP3$^{-/-}$ mice were co-cultured for 24 hr with macrophages from CCl$_4$-injected WT or AQP3$^{-/-}$ mice, corresponding to Fig. 3f. Expression of TGF-β1 mRNA in HSCs (mean±SE, n=5 biologically independent samples, **p < 0.01 by one-way ANOVA with Dunnett's multiple comparisons test). Source data, including exact p values, are provided as a Source Data file.

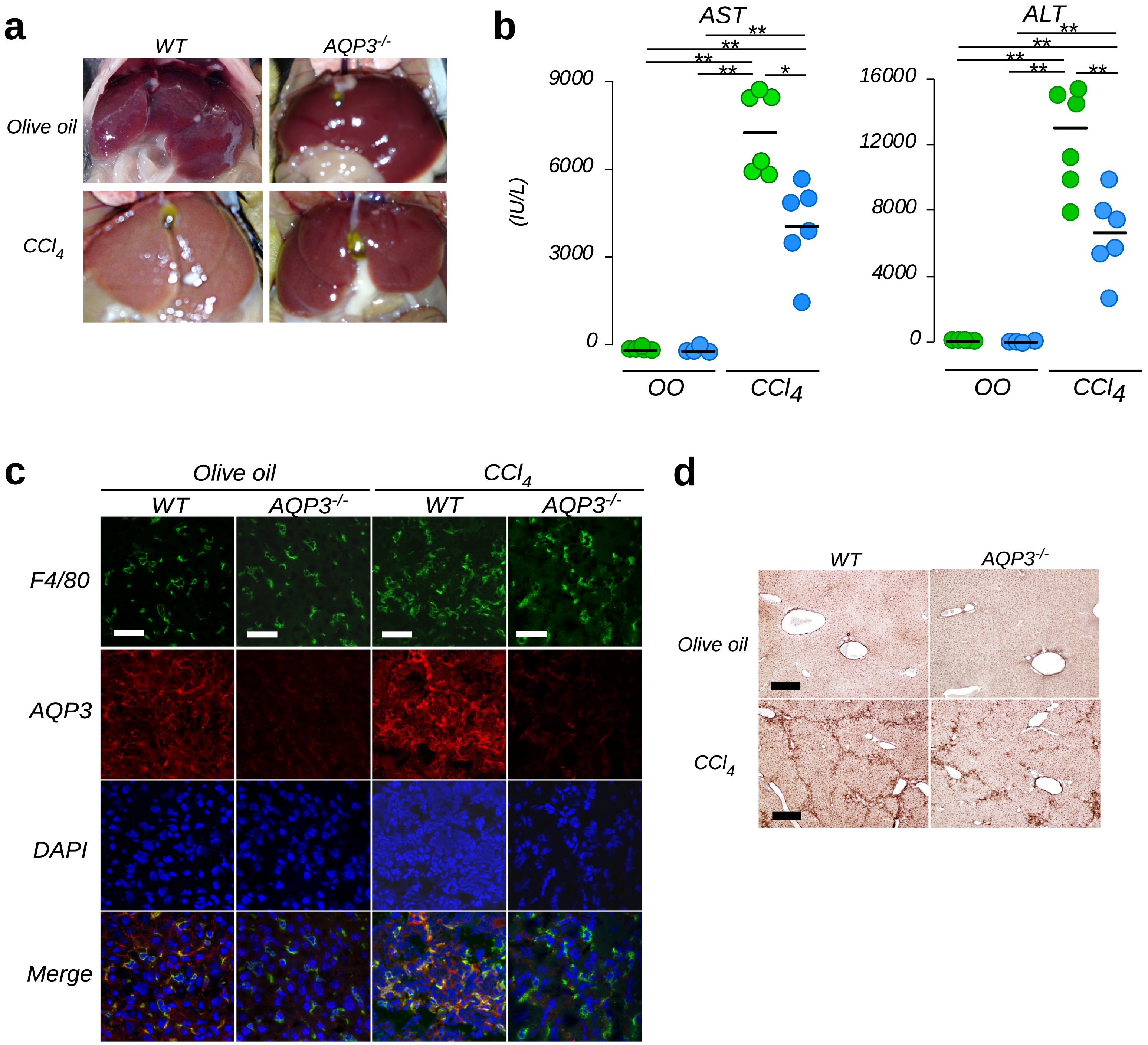

Supplementary Figure 4

a. Representative photographs of the liver following CCl_4 injection for six weeks. **b.** Serum AST and ALT corresponding to Fig. 4a-4g. (mean±SE, n=4 for olive oil, n=6 for CCl_4 mice/group, *$p < 0.05$, **$p < 0.01$ by two-way ANOVA with Tukey's multiple comparisons test) **c.** Immunostaining with anti-AQP3 (cy3, red) and anti-F4/80 (Alexa 488, green) in mouse liver, corresponding to Fig. 4a-4g (DAPI, blue). Scale bar, 100 µm. **d.** Immunostaining of α-SMA in WT and $AQP3^{-/-}$ mouse liver corresponding to Fig. 4a-4g. Bar, 200 µm. Source data, including exact p values, are provided as a Source Data file.

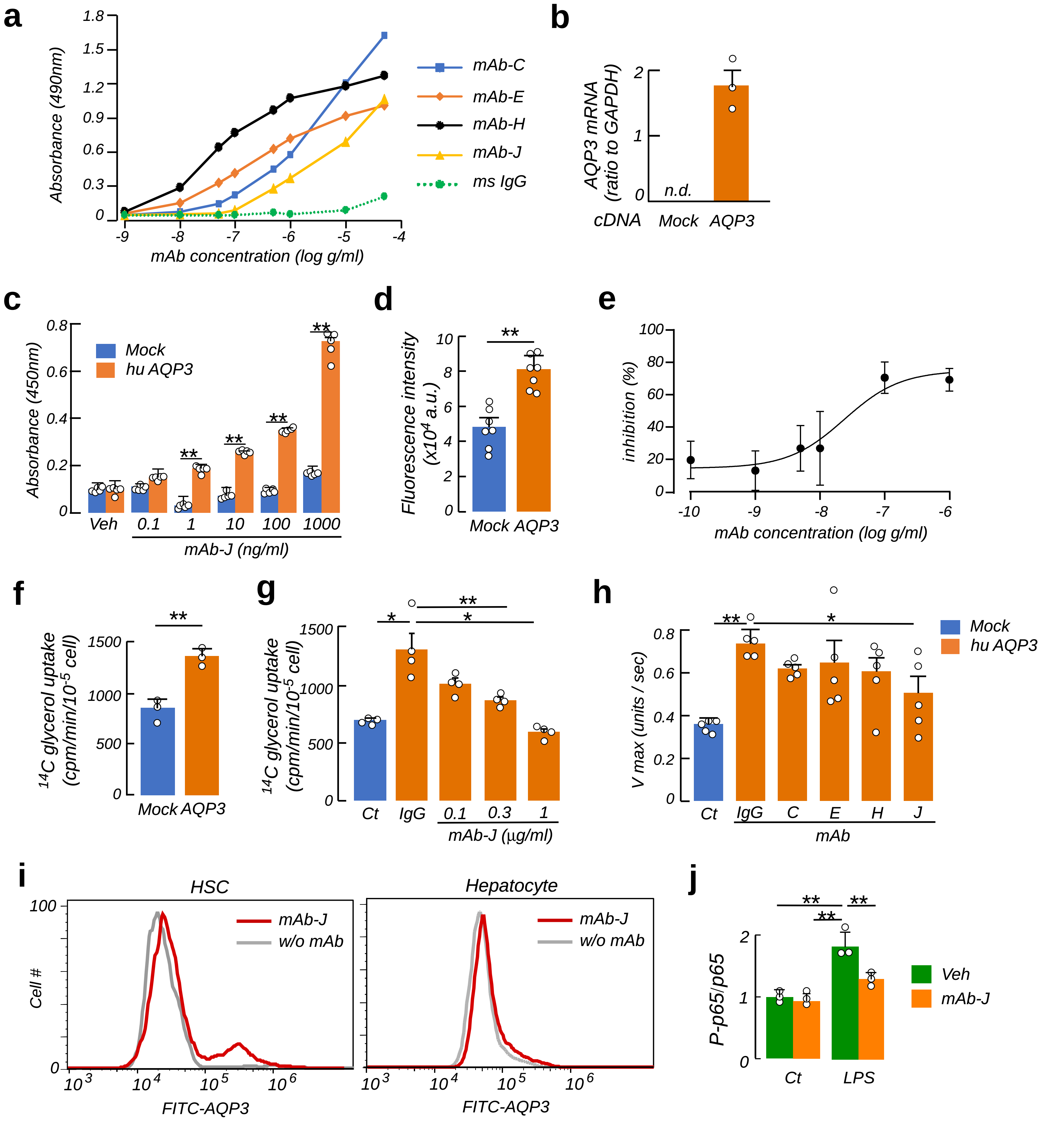

Supplementary Figure 5

a. Binding of the mAbs (C, E, H, J) and mouse IgG (control) to the oligopeptide with amino acid sequence corresponding to positions 148 to 157 of the mouse/human AQP3 protein. Experiments were performed in duplicate and repeated three times with similar results. **b-h.** CHO-K1 cells were transfected with pCMV6 empty vector (mock) or plasmid encoding human AQP3. **b.** Relative mRNA expression of AQP3/GAPDH (mean±SE, n=3 biologically independent samples). **c.** Binding of mAb-J to human AQP3-expressing CHO-K1 and mock cells (mean±SE, n=5 biologically independent samples, $^{**}p < 0.01$ by two-tailed unpaired Student t-test). **d.** Increased H_2O_2 uptake in human AQP3-expressing CHO-K1 cells compared with mock cells. Cellular H_2O_2 measured by CM-H_2DCFDA fluorescence after adding H_2O_2 (60 µM) (mean±SE, n=7 biologically independent samples, $^{**}p < 0.01$ by two-tailed unpaired Student t-test). **e.** Inhibition of H_2O_2 uptake in human AQP3-expressing CHO-K1 cells by mAb-J (mean±SE, n=8 biologically independent samples). **f.** Increased glycerol uptake as measured using [^{14}C]-labeled glycerol in human AQP3-expressing CHO-K1 cells compared with mock cells (mean±SE, n=3 biologically independent samples, $^{**}p < 0.01$ by two-tailed unpaired Student t-test). **g.** Inhibition of glycerol uptake in human AQP3-expressing CHO-K1 cells by mAb-J (mean±SE, n=4 biologically independent samples, $^{*}p < 0.05$, $^{**}p < 0.01$ by one-way ANOVA with Dunnett's multiple comparisons test). **h.** Osmotic water permeability in human AQP3-expressing CHO-K1 cells. Cells were incubated with mAb (1 µg/ml) for 1hr and stained with calcein AM (10 µM, 15 min, Invitrogen). Osmotic cell shrinkage was induced by addition of 1 volume of 600 mM sucrose in PBS, and the fluorescence intensity of calcein fluorescence quenching was recorded by plate reader (Ex, 492nm, Em, 525 nm, SpectraMax i3x; Molecular Devices). V max was calculated with GraphPad Prism8 (mean±SE, n=5 biologically independent samples, $^{*}p < 0.05$, $^{**}p < 0.01$ by one-way ANOVA with Dunnett's multiple comparisons test). **i.** Flow cytometric analysis of binding of anti-AQP3 mAb-J to WT HSC or hepatocytes. **j.** Ratio of P-p65 to p65, from data as in Fig. 5g (mean±SE, n=3 biologically independent samples, $^{**}p < 0.01$ by two-way ANOVA). Source data, including exact p values, are provided as a Source Data file.

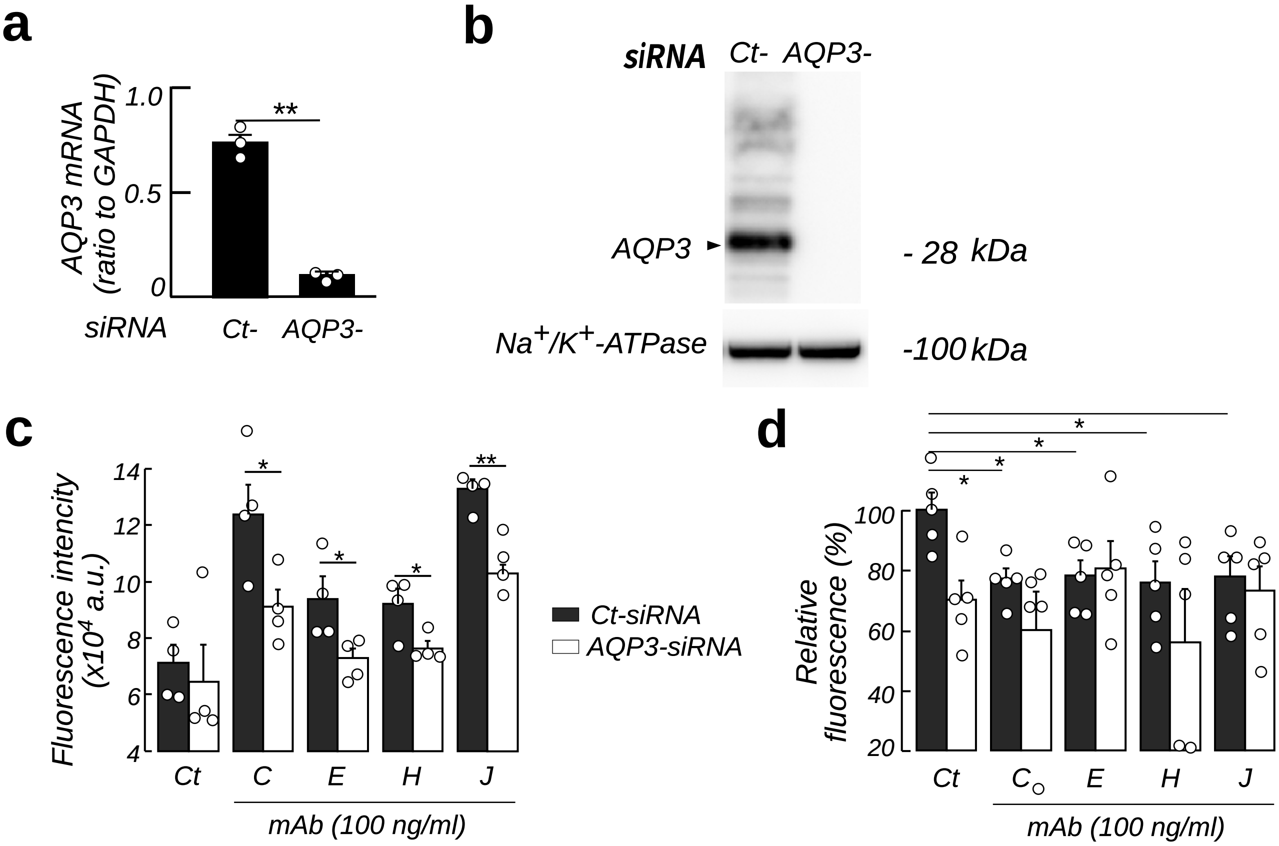

Supplementary Figure 6

a-d. HaCaT cells were transfected with human AQP3- or non-targeting (Ct)-siRNA. **a.** Relative mRNA expression of AQP3/GAPDH (mean±SE, n=3 biologically independent samples, $**p < 0.01$ by two-tailed unpaired Student t-test). **b.** Immunoblot of membrane fraction with anti-AQP3 or anti-Na^+ /K^+ ATPase antibodies. **c.** Binding of mAb to HaCaT cells (mean±SE, n=4 biologically independent samples, $*p < 0.05$, $**p < 0.01$ by two-tailed unpaired Student t-test). **d.** Effect of mAb on H_2O_2 uptake in HaCaT cells. Cellular H_2O_2 measured using CM-H_2DCFDA fluorescence after adding H_2O_2 (30 μM) (mean±SE, n=5 biologically independent samples, $*p < 0.05$ by one-way ANOVA). Source data, including exact p values and uncropped immunoblot image, are provided as a Source Data file.

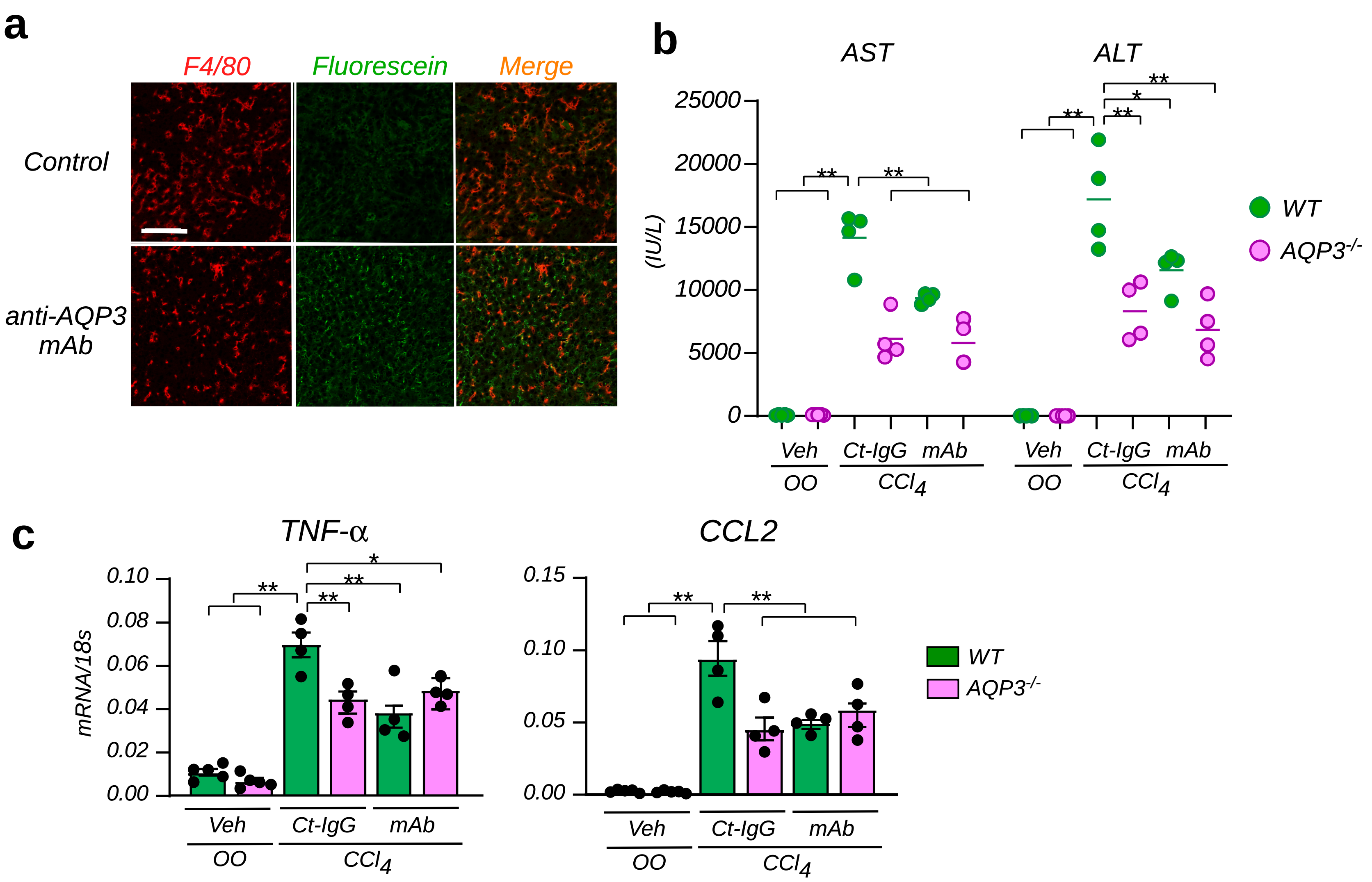

Supplementary Figure 7

a. Immunofluorescence (green) of liver after injection with anti-AQP3 mAb-conjugated fluorescein. Liver was excised at 6 or 24 hours after injection, and frozen section was stained with F4/80 (Cy3, red). Representative immunofluorescence at 6 hr. Bar, 200 µm. **b-c.** Anti-AQP3 (mAb, 10 mg/kg body weight, PBS) or control monoclonal anti-mouse IgG (Ct-IgG, 10 mg/kg body weight, PBS) was injected intravenously into WT and AQP3$^{-/-}$ mice one day before CCl_4 injection (1 ml/kg body weight, olive oil; OO). Liver and blood were collected at 24 hr. **b.** Serum AST and ALT (mean±SE, n=4 for CCl_4, n=5 for olive oil mice/group, *p < 0.05, **p < 0.01 by two-way ANOVA with Tukey's multiple comparisons test). **c.** mRNA expression of indicated genes by real-time RT-PCR. Data are expressed as the ratio to 18s (mean±SE, n=4 for CCl_4, n=5 for olive oil mice/group, *p < 0.05, **p < 0.01 by two-way ANOVA with Tukey's multiple comparisons test). Source data, including exact p values, are provided as a Source Data file.

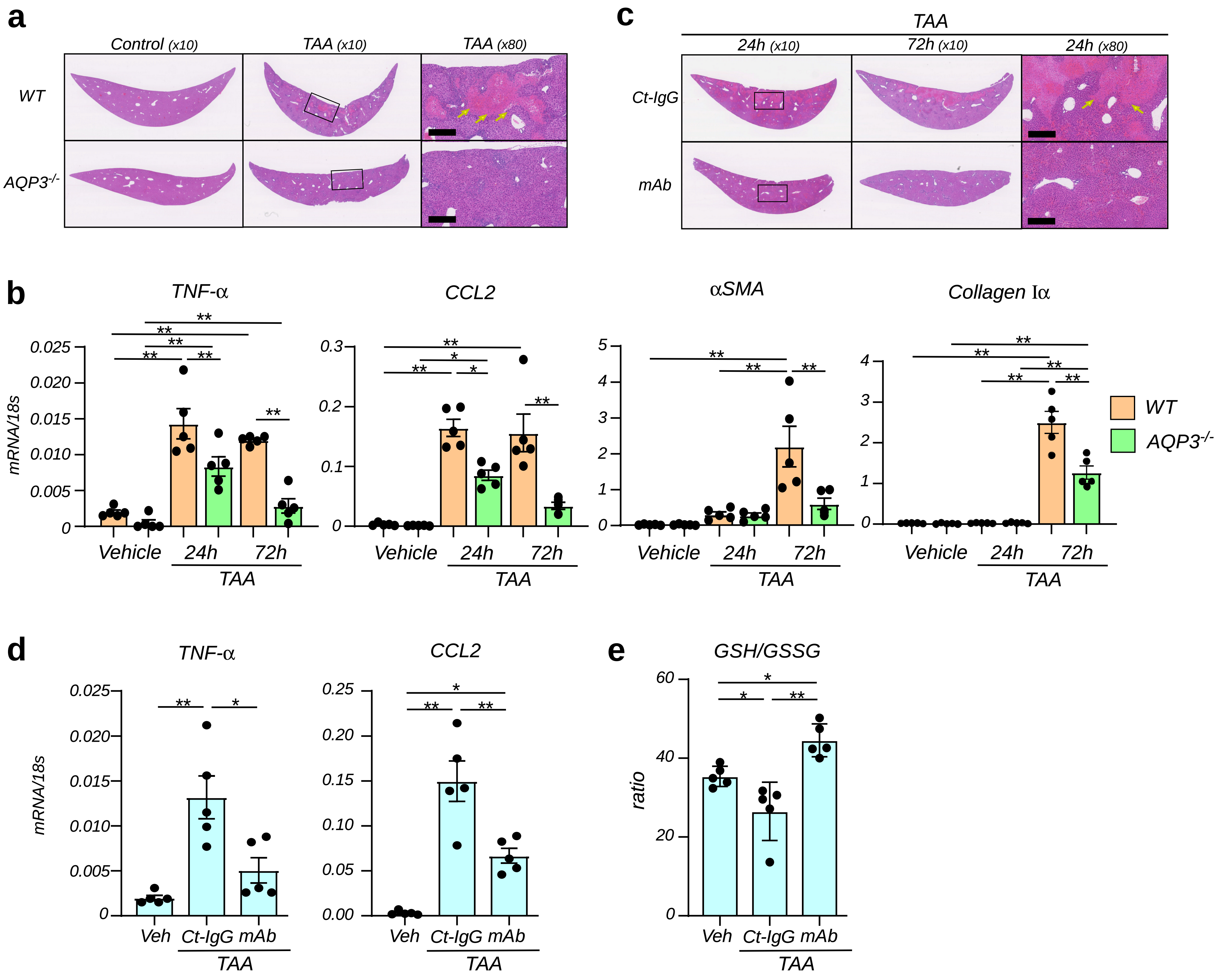

Supplementary Figure 8

Mouse model of TAA-induced liver injury. **a. b.** WT or AQP3$^{-/-}$ mice received an intraperitoneal injection of TAA (thioacetamide, 200 μg/g body weight) or vehicle (saline). Mice were sacrificed at 24 or 72 hours after TAA injection. **a.** Hematoxylin and eosin staining of livers from WT and AQP3$^{-/-}$ mice at 72 hr. Bar, 200 μm. **b.** mRNA expression of indicated genes in liver homogenates by real-time RT-PCR (mean±SE, n=5 mice/group, $*p < 0.05$, $**p < 0.01$). Data are expressed as the ratio to 18s RNA. **c.-e.** Effect of anti-AQP3 mAb on TAA-induced acute liver injury. Anti-AQP3 mAb (mAb-J, 10 mg/kg body weight, PBS) or mouse monoclonal antibody (as a control IgG) was administered intravenously (10 mg/kg) 1 day before each TAA injection. **c.** Hematoxylin and eosin staining of liver from WT and AQP3$^{-/-}$ mice. Bar, 200 μm. **d.** mRNA expression of indicated genes in liver homogenates by real-time RT-PCR (mean±SE, n=5 mice/group, $*p < 0.05$, $**p < 0.01$). Data are expressed as the ratio to 18s RNA. **e.** Ratio of GSH to GSSG in liver homogenate (mean±SE, n=5 mice/group, $*p < 0.05$, $**p < 0.01$). Statistical analysis for Supplementary Fig. 8b, 8d, and 8e was performed by two-way ANOVA with Tukey's multiple comparisons test. Source data, including exact p values, are provided as a Source Data file.

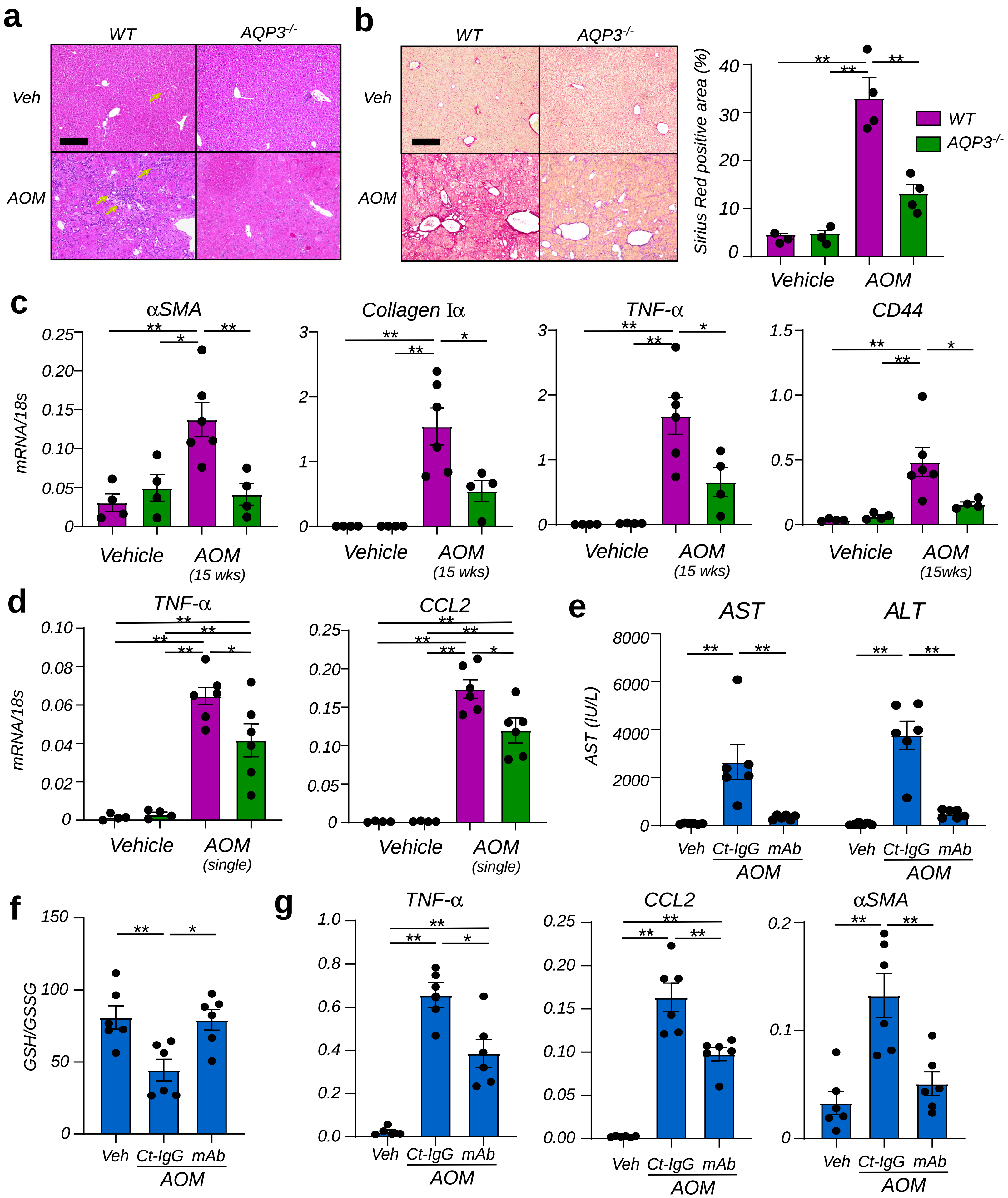

Supplementary Figure 9

AOM-induced acute and chronic liver injury model. **a-c.** AOM (azoxymethane, 10 μg/ g body weight) or vehicle (saline) was intraperitoneally injected once weekly for 6 weeks and mice were sacrificed at 15 weeks. **a.** Hematoxylin and eosin staining of liver from WT and AQP3$^{-/-}$ mice. Bar, 200 μm. Arrow; infiltrating lymphocytes. **b.** (left) Sirius red staining. Bar, 200 μm. (right) % Areas of Sirius red staining (mean±SE, n=3 for vehicle, n=4 for AOM sections from individual mice, **p < 0.01). **c.** mRNA expression of indicated genes in liver homogenates by real-time RT-PCR (mean±SE, n=4; WT/AQP3$^{-/-}$ with vehicle and AQP3$^{-/-}$with AOM, n=6; WT with AOM mice/group, *p < 0.05, **p < 0.01). Data are expressed as the ratio to 18s RNA. **d.** AOM (30 μg/g body weight) or vehicle (saline) was injected intraperitoneally, and livers were excised at 24 hr. mRNA expression of indicated genes by real-time RT-PCR (mean ±SE, n=4 for vehicle, n=6 for AOM, *p < 0.05, **p < 0.01). Data are expressed as the ratio to 18s. **e-g.** Effect of anti-AQP3 mAb on AOM-induced acute liver injury. Anti-AQP3 (mAb-J, 10 mg/kg body weight, PBS) or control monoclonal anti-mouse IgG (Ct-IgG, 10 mg/kg body weight, PBS) was injected intravenously 1 day before AOM injection (30 μg/g body weight). Liver and blood were collected at 24 hr. **e.** Serum AST and ALT. **f.** Ratio of GSH to GSSG in liver homogenate. **g.** mRNA expression of indicated genes by real-time RT-PCR. Data are expressed as the ratio to 18s (mean±SE, n=6 mice/group, *p < 0.05, **p < 0.01). Statistical analysis for Sup Fig. 9b-g was performed by two-way ANOVA with Tukey's multiple comparisons test. Source data, including exact p values, are provided as a Source Data file.

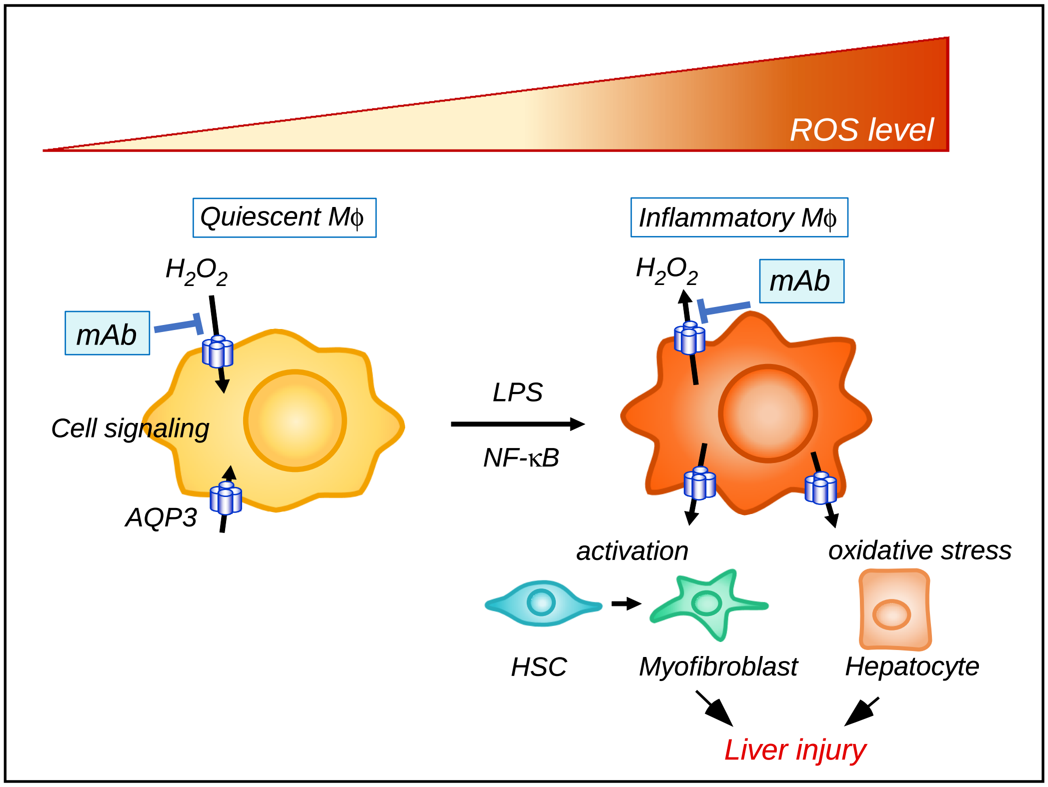

Supplementary Figure 10

Schematic showing proposed mechanism of AQP3-mediated H_2O_2 transport in hepatic macrophages during liver injury. In quiescent macrophages, AQP3 transported-H_2O_2 is involved as a second messenger in NF-κB activation. In inflammed macrophages, AQP3 releases H_2O_2 into the extracellular space, resulting in HSC activation and oxidative stress. Anti-AQP3 mAb inhibits H_2O_2 cellular influx and efflux.
