# Supplementary material for: Inhibition of aquaporin-3 in macrophages by a monoclonal antibody as potential therapy for liver injury
Source: Nat Commun. 2020 Nov 9;11:5666. doi: 10.1038/s41467-020-19491-5 (PMC7653938; doi:10.1038/s41467-020-19491-5)
Supplement: Supplementary file 3 — Reporting Summary [file 41467_2020_19491_MOESM3_ESM.pdf]

nature research

Corresponding author(s): Mariko Hara-Chikuma

Last updated by author(s): 2020/9/10

Reporting Summary

Nature Research wishes to improve the reproducibility of the work that we publish. This form provides structure for consistency and transparency in reporting. For further information on Nature Research policies, see Authors & Referees and the Editorial Policy Checklist .

Statistics

For all statistical analyses, confirm that the following items are present in the figure legend, table legend, main text, or Methods section.

n/a	Confirmed	
☐	☒	The exact sample size (*n*) for each experimental group/condition, given as a discrete number and unit of measurement
☐	☒	A statement on whether measurements were taken from distinct samples or whether the same sample was measured repeatedly
☐	☒	The statistical test(s) used AND whether they are one- or two-sided<br>*Only common tests should be described solely by name; describe more complex techniques in the Methods section.*
☐	☒	A description of all covariates tested
☒	☐	A description of any assumptions or corrections, such as tests of normality and adjustment for multiple comparisons
☐	☒	A full description of the statistical parameters including central tendency (e.g. means) or other basic estimates (e.g. regression coefficient) AND variation (e.g. standard deviation) or associated estimates of uncertainty (e.g. confidence intervals)
☐	☒	For null hypothesis testing, the test statistic (e.g. *F*, *t*, *r*) with confidence intervals, effect sizes, degrees of freedom and *P* value noted<br>*Give P values as exact values whenever suitable.*
☒	☐	For Bayesian analysis, information on the choice of priors and Markov chain Monte Carlo settings
☒	☐	For hierarchical and complex designs, identification of the appropriate level for tests and full reporting of outcomes
☒	☐	Estimates of effect sizes (e.g. Cohen's *d*, Pearson's *r*), indicating how they were calculated

Our web collection on statistics for biologists contains articles on many of the points above.

Software and code

Policy information about availability of computer code

Data collection	No software was used for data collection
Data analysis	GraphPad Prism8, FlowJo for flow cytometry analysis, Tissue quest and Image J for image analysis,

For manuscripts utilizing custom algorithms or software that are central to the research but not yet described in published literature, software must be made available to editors/reviewers. We strongly encourage code deposition in a community repository (e.g. GitHub). See the Nature Research guidelines for submitting code & software for further information.

Data

Policy information about availability of data

All manuscripts must include a data availability statement. This statement should provide the following information, where applicable:

- Accession codes, unique identifiers, or web links for publicly available datasets
- A list of figures that have associated raw data
- A description of any restrictions on data availability

All data supporting the findings of this study are available from the corresponding authors on reasonable request.

Field-specific reporting

Please select the one below that is the best fit for your research. If you are not sure, read the appropriate sections before making your selection.

☒ Life sciences ☐ Behavioural & social sciences ☐ Ecological, evolutionary & environmental sciences

For a reference copy of the document with all sections, see nature.com/documents/nr-reporting-summary-flat.pdf

Life sciences study design

All studies must disclose on these points even when the disclosure is negative.

Sample size	The chosen sample size are based on the numbers used for previous publications, which is most optimal to generate statistically significant results.
Data exclusions	No data were excluded from the analysis.
Replication	For in vitro study, at least 2 independent sets were performed, and all findings were reliably reproduced.
Randomization	No randomization was applied in this study
Blinding	In vivo experiments using mice were performed in a blinded fashion such that the people conducting and/or analyzing the assay were not aware of treatment groups or mice gene until data gathering were complete.

Reporting for specific materials, systems and methods

We require information from authors about some types of materials, experimental systems and methods used in many studies. Here, indicate whether each material, system or method listed is relevant to your study. If you are not sure if a list item applies to your research, read the appropriate section before selecting a response.

Materials & experimental systems

n/a	Involved in the study	
☐	☒	Antibodies
☐	☒	Eukaryotic cell lines
☒	☐	Palaeontology
☐	☒	Animals and other organisms
☒	☐	Human research participants
☒	☐	Clinical data

Methods

n/a	Involved in the study	
☒	☐	ChIP-seq
☐	☒	Flow cytometry
☒	☐	MRI-based neuroimaging

Antibodies

Antibodies used

anti-mouse F4/80 eBioscience 14-4801
anti-Desmin R&D systems AF6844
anti-phospho-p65 Cell signaling technology 3033
anti-NF-kappaB p65 Cell signaling technology 8242
anti-mouse CD11B-APC eBioscience 17-0112
anti-mouse F4/80-PE Cy7 eBioscience 25-4801
anti-mouse F4/80 eBioscience 14-4801-82
anti-AQP3 Millopore AB3276
goat anti-rabbit IgG-FITC sigma F9887
phospho-H2A.X Cell signaling technology 9718
anti-TNF-alpha fluoro 450 eBioscience 48-7321
anti-CD45.1 PE-Cy7 Invitrogen 25-0453-82
anti-CD45.2 APC eBioscience 17-0454-81
anti-Na-K-ATPase Millipore 05-369
anti-smooth muscle actin DAKO M0851
anti-CD68 Proteintech 66231-2-Ig

Validation

Antibody specificity was assessed by the manufacturer. All antibodies were titrated prior to use.

Eukaryotic cell lines

Policy information about cell lines

Cell line source(s)	CHO-K1 cells (Riken BRC cell bank, JAPAN)<br>HaCaT cells (from Dr Fusenig, German Cancer Research Center, Heidelberg, Germany)
Authentication	All cell lines were authenticated using the short tandem repeat profiling.

Mycoplasma contamination	All cell lines were periodically tested for mycoplasma contamination using MycoAlert™ mycoplasma detection kit (Lonza). None of the cell lines were contaminated.
Commonly misidentified lines (See ICLAC register)	No commonly misidentified cell lines were used in this study.

Animals and other organisms

Policy information about studies involving animals; ARRIVE guidelines recommended for reporting animal research

Laboratory animals	C57BL/6 mice , 7-10 weeks old, AQP3 knockout mice, 7-10 weeks old
Wild animals	The study did not involve wild animals.
Field-collected samples	The study did not involve samples collected from the field.
Ethics oversight	All research involving animal experiments were approved by the Committee on Animal Research of Keio University

Note that full information on the approval of the study protocol must also be provided in the manuscript.

Flow Cytometry

Plots

Confirm that:

- [x] The axis labels state the marker and fluorochrome used (e.g. CD4-FITC).
- [x] The axis scales are clearly visible. Include numbers along axes only for bottom left plot of group (a 'group' is an analysis of identical markers).
- [x] All plots are contour plots with outliers or pseudocolor plots.
- [x] A numerical value for number of cells or percentage (with statistics) is provided.

Methodology

Sample preparation	Livers were perfused with liver perfusion medium (Invitrogen) and then liver digest medium (Invitrogen). For Fig. 1d, cell suspension were stained with anti-F4/80 and anti-CD11B. For Fig 2F, the dispersed cells were centrifuged (50x g, 1 minute) to remove hepatocytes as pellets. The recovered nonparenchymal cells containing macrophages were stained with anti-F4/80 and anti-CD11B, and incubated by Fixation/permeabilization solution (BD bioscience). Cells were incubated with BD perm/wash buffer (BD bioscience), following stained with anti-TNF-alpha.
Instrument	Gallios (Beckman)
Software	FlowJo
Cell population abundance	Approximately 10 % of cells were determined for each.
Gating strategy	For Fig. 2f, CD11B+ F4/80+ cells were collected.

- [ ] Tick this box to confirm that a figure exemplifying the gating strategy is provided in the Supplementary Information.
